# Quantitative Assessment of Common Genetic Variants on Chromosome 5p12 and Hormone Receptor Status with Breast Cancer Risk

**DOI:** 10.1371/journal.pone.0072154

**Published:** 2013-08-19

**Authors:** Yanmin Yu, Zenggan Chen, Hong Wang, Yan Zhang

**Affiliations:** 1 Department of Breast Surgery, Huangpu Central Hospital of Shanghai, Shanghai, People’s Republic of China; 2 Department of Orthopedics, Zhongshan Hospital, Fudan University, Shanghai, People’s Republic of China; 3 Department of General Surgery, Zhongshan Hospital, Fudan University, Shanghai, People’s Republic of China; 4 Department of Radiology, Huangpu Central Hospital of Shanghai, Shanghai, People’s Republic of China; MOE Key Laboratory of Environment and Health, School of Public Health, Tongji Medical College, Huazhong University of Science and Technology, China

## Abstract

Several genome-wide association studies on breast cancer (BC) have reported similar findings of a new susceptibility locus, 5p12. After that, a number of studies reported that the rs10941679, rs4415084, and rs981782 polymorphism in chromosome 5p12 has been implicated in BC risk. However, the studies have yielded contradictory results. To derive a more precise estimation of the relationship, a meta-analysis of 131,983 BC cases and 200,314 controls from 24 published case–control studies was performed. Overall, significantly elevated BC risk was associated with rs10941679, rs4415084, and rs981782 risk allele when all studies were pooled into the meta-analysis. In the subgroup analysis by ethnicity, significantly increased risks were found for the rs10941679 and rs4415084 polymorphism among Caucasians and East Asians, while no significant associations were observed for the two polymorphisms in African and other ethnic populations. For 5p12-rs981782, significant associations were only detected among Caucasians. In addition, we found that rs10941679 and rs4415084 on 5p12 confer risk, exclusively for estrogen receptor (ER)-positive tumors with per-allele OR of 1.16 (95% CI: 1.11–1.21; P<10^−5^) and of 1.14 (95% CI: 1.09–1.19; P<10^−5^) respectively. Ethnicity was identified as a potential source of between-study heterogeneity. In conclusion, this meta-analysis demonstrated that common variations are a risk factor associated with increased BC susceptibility, but these associations vary in different ethnic populations.

## Introduction

Breast cancer (BC) is one of the most common malignancies among women worldwide [1]. Although life/environment related factors, such as age at menarche, menopause and first birth and exogenous hormone use are implicated in breast carcinogenesis [2], [3], it is a complex polygenic disorder for which genetic factors play an important role in disease etiology [4], [5]. In the past decades, high-penetrance genes (for example, *BRCA1*, *BRCA2*, *PTEN* and *TP53*) have been identified to be associated with familiar breast cancer [6]. However, these genes account for less than 5% of overall breast cancer patients and most of the risk is likely to be attributable to more low-penetrance genetic variants [7], [8].

Recently, spectacular advance was made in identifying susceptible genes involved in breast cancer through genome-wide association strategy (GWAS) [9]–[11]. So far, genome-wide association studies (GWASs) have reported over 40 common low-penetrance variants in 25 loci that are associated with the breast cancer risk reported in the National Human Genome Research Institute catalog [12]. The most strongly and consistently associated single-nucleotide polymorphisms (SNPs) reside in intron 2 of the receptor tyrosine kinase *FGFR2* (rs2981582) at 10q26.13 and near the 5′ end of the *TOX3* gene at 16q12.1 (rs3803662) [13], [14]. The chromosome 5p12 region has been identified as a hotspot for breast cancer susceptibility by recent genome-wide association (GWA) studies [15], [16]. Three polymorphisms (rs10941679, rs4415084, and rs981782) in the region and breast cancer risk have been independently replicated by subsequent studies; however, a proportion of them have produced contrary results. These disparate findings may be due partly to insufficient power, phenotypic heterogeneity, population stratification, small effect of the polymorphism on breast cancer risk, and even publication biases. In addition, with the increased studies in recent years among Asian and African populations, there is a need to reconcile these data. We therefore performed a meta-analysis of the published studies to clarify this inconsistency and to establish a comprehensive picture of the relationship between common variants on chromosome 5p12 and breast cancer.

## Materials and Methods

### Literature Search Strategy and Selection Criteria

Genetic association studies published before the end of March 2013 on breast cancer and polymorphisms in the chromosome 5p12 were sought by computer-based searches from databases including MEDLINE, PubMed, EMBASE, ISI web of science and CNKI (China National Knowledge Infrastructure) without language restriction. Search term combinations were keywords relating to the chromosome 5p12 (e.g., “5p12”, “rs10941679”, “rs4415084”, “rs981782”) in combination with words related to breast cancer (e.g., breast cancer’ or ‘malignant breast neoplasm). We replaced one term each time until all possible combination mode were searched to avoid any missing literature. The titles and abstracts of potential articles were screened to determine their relevance, and any clearly irrelevant studies were excluded. The full texts of the remaining articles were read to determine whether they contained information on the topic of interest. Furthermore, reference lists of primary studies and review articles were also reviewed by a manual search to identify additional relevant publications.

The included studies have to meet the following criteria: (1) original papers containing independent data, (2) identification of breast cancer patients was confirmed histologically or pathologically, (3) genotype distribution information or odds ratio (OR) with its 95% confidence interval (CI) and P-value, (4) case–control or cohort studies. The major reasons for exclusion of studies were (1) overlapping data, (2) case-only studies, (3) family based studies, and review articles.

### Data Extraction

Data extraction was performed independently by two reviewers and differences were resolved by further discussion among all authors. For each included study, the following information was extracted from each report according to a fixed protocol: first author, publication year, definition and numbers of cases and controls, frequency of genotypes, age, cigarette smoking, alcohol drinking, ethnicity, Hardy–Weinberg equilibrium (HWE) status, source of control, estrogen receptor (ER) status, progesterone receptor (PR) status and genotyping method. Studies with different ethnic groups were considered as individual studies for our analyses.

### Statistical Methods

Deviation from HWE for controls was examined by χ2 tests. OR with 95% CIs was used to assess the strength of association between the 5p12 polymorphisms and breast cancer risk. The per-allele OR of the risk allele was estimated. Then we estimated the risks of the heterozygous and homozygote genotypes on breast cancer, compared with the wild-type homozygote. Random-effects and fixed-effect summary measures were calculated as inverse-variance-weighted average of the log odds ratio. The results of random-effects summary were reported in the text because it takes into account the variation between studies. Heterogeneity across individual studies was calculated using the Cochran χ^2^ based Q test followed by subsidiary analysis or by random-effects regression models with restricted maximum likelihood estimation [17], [18]. Sources of heterogeneity were investigated by stratified meta-analyses based on ethnicity, and ER status. Ethnic group was defined as East Asians (i.e., Chinese, Japanese, and Korean), Caucasians (i.e. people of European origin), Africans and other ethnic populations. In addition, ethnicity, sample size, age at test and genotyping method was analyzed as covariates in meta-regression. The significance of the pooled OR was determined by Z test. Publication bias was assessed with the Egger test and Begg test [19], [20]. Sensitivity analysis was performed by removing each individual study in turn from the total and re-analyzing the remainder. Power analysis was calculated with the pooled OR estimate from different ethnicity and allele frequency in controls. The analysis was conducted using the Stata software version 10.0 (Stata Corporation, College Station, TX). All the P-values were for two-sided analysis and values of P<0.05 were considered statistically significant.

## Results

### Study Characteristics

The combined search yielded 109 references. Figure S1 shows the study selection process. Finally, a total of 24 eligible association studies with 131,983 BC cases and 200,314 controls were identified [9], [15], [16], [21]–[41], with 10 studies genotyping more than one variant. The main study characteristics were summarized in Table 1. There are 31 data sets from 15 studies with 85,279 BC cases and 144,552 controls concerning rs10941679 and 32 data sets from 14 studies involving 40,446 BC cases and 74,403 controls concerning rs4415084. For the rs981782 polymorphism, 17 data sets from 7 studies involved a total of 44,609 BC cases and 78,192 controls. These three polymorphisms were found to occur in frequencies consistent with HWE in the control populations of the all published studies. Statistical power to detect risk allele is 96%, 92% and 90% for rs10941679, rs4415084 and rs981782 polymorphism, respectively.

**Table 1:** Characteristics of the studies included in the meta-analysis.

Study	Year	Ethnicity	Genotyping method	No. of cases/controls	Control source
Easton [9]	2007	European, America, Australian	SNP Array, TaqMan	15335/17802	GP
Stacey [15]	2008	European	SNP Array	5028/32090	GP
Thomas [16]	2009	American, European	SNP Array, TaqMan	7849/9835	GP
Mcinerney [21]	2009	Irish	KASPar	882/997	GP
Zheng [22]	2009	African American	Massarray	810/1784	GP
Ruiz-Narvaez [23]	2010	African American	iPLEX	886/1089	GP
Antoniou [24]	2010	European, America	iPLEX	8534/7011	GP
Bhatti [25]	2010	American	TaqMan	776/997	GP
Reeves [26]	2010	British	TaqMan	10306/10393	GP
Zheng [27]	2010	Chinese	SNP Array	3039/3082	GP
Barnholtz-Sloan [28]	2010	American	GoldenGate	1971/1775	GP
Wang [29]	2010	European, American	SNP Array	3030/2427	GP
Teraoka [30]	2011	Dane, American	Golden Gate	704/1387	GP
Fletcher [31]	2011	British	SNP Array, GoldenGate	7643/7443	GP
Campa [32]	2011	American, European	TaqMan	6396/9225	GP
Milne [33]	2011	European, North America, Australian, Asian	TaqMan	45377/74253	GP
Li [34]	2011	European	SNP Array	1557/4584	GP
Kim [35]	2012	Korean	SNP Array, TaqMan	2257/2052	GP
Huo [36]	2012	Nigerian	GoldenGate	1509/1383	GP
Chan [37]	2012	Chinese	TaqMan	1175/1499	GP
Sueta [38]	2012	Japanese	TaqMan	697/1394	HP
Liu [39]	2012	Chinese	TaqMan	846/882	GP
Harlid [40]	2012	European	MassARRAY	3584/5063	GP
Dai [41]	2012	Chinese	TaqMan	1792/1867	GP

GP: general population, HP: hospital patient.

### Association of rs10941679 Polymorphism with Breast Cancer

Overall, there was evidence of an association between the increased risk of BC and the variant in different genetic models when all the eligible studies were pooled into the meta-analysis (Figure S2). Using random effect model, significantly increased risks were found among Caucasian populations (G allele: OR = 1.08, 95% CI: 1.05–1.12, P<10^−5^; heterozygous: OR = 1.10, 95% CI: 1.06–1.15, P<10^−5^; homozygote: OR = 1.12, 95% CI: 1.05–1.20, P<10^−5^). Similar significant associations were also observed for East Asians with per-allele OR of 1.05 (95% CI: 1.01–1.09, P = 0.01). However, no significant association was found for African and other ethnic populations in all genetic models (Table 2).

**Table 2:** Meta-analysis of the 5p12 rs10941679 polymorphism on breast cancer risk.

Sub-groupanalysis	No. of data sets	No. of cases/controls	G allele				Heterozygous				Homozygous			
			OR (95%CI)	P(Z)	P(Q)^a^	P(Q)^b^	OR (95%CI)	P(Z)	P(Q)^a^	P(Q)^b^	OR (95%CI)	P(Z)	P(Q)^a^	P(Q)^b^
Ethnicity						0.02				0.008				0.04
Caucasian	18	70103/127620	1.10 (1.06–1.15)	<10^−5^	<10^−4^		1.11 (1.05–1.16)	<10^−5^	0.002		1.15 (1.03–1.28)	0.01	<10^−4^	
East Asian	7	11093/11588	1.05 (1.01–1.09)	0.01	0.63		1.05 (0.98–1.12)	0.20	0.59		1.09 (1.02–1.18)	0.01	0.87	
African	4	3569/4658	1.01 (0.93–1.10)	0.81	0.37		1.12 (0.92–1.35)	0.27	0.001		1.01 (0.76–1.35)	0.95	0.93	
Other	2	514/686	1.18 (0.99–1.40)	0.07	0.51		1.20 (0.93–1.56)	0.17	0.70		1.45 (1.01–2.09)	0.05	0.36	

a Cochran’s chi-square Q statistic test used to assess the heterogeneity in subgroups.

b Cochran’s chi-square Q statistic test used to assess the heterogeneity between subgroups.

**Figure 1:**
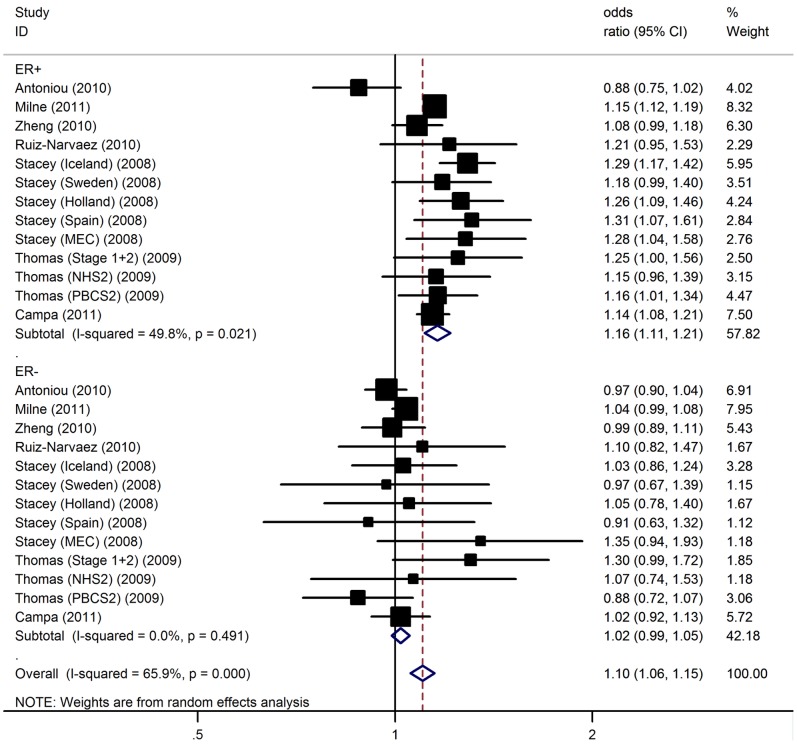
Association between 5p12 rs10941679 and breast cancer risk by ER status.

Furthermore, we performed case–control analyses by subgroups according to estrogen receptor status. A stronger association was observed for the polymorphism with ER-positive tumors [per-allele OR = 1.16, 95% CI: 1.11–1.21; P(Z) <10^−5^; P(Q) = 0.02] versus ER-negative tumors [per-allele OR = 1.02, 95% CI: 0.99–1.05; P(Z) = 0.21; P(Q) = 0.49] (Figure 1).

Significant heterogeneity was present among the 33 data sets (P<0.05). In meta-regression analysis, sample size (P = 0.13), genotyping method (P = 0.37), mean age of cases (P = 0.25) and controls (P = 0.61), did not significantly explained such heterogeneity. By contrast, ethnicity (P = 0.001) was significantly correlated with the magnitude of the genetic effect.

### Association of rs4415084 Polymorphism with Breast Cancer

Significant heterogeneity was present among the included studies of the rs4415084 polymorphism (P<0.05). Ethnicity (P = 0.007) and sample size (P = 0.03) explained a large part of the heterogeneity, whereas genotyping method (P = 0.58), mean age of cases (P = 0.18) and controls (P = 0.32) explained little heterogeneity. In the overall analysis, the rs4415084 polymorphism on chromosome 5p12 was significantly associated with elevated BC risk for Caucasians with a per-allele OR of 1.10 (95% CI: 1.06–1.14, P<10^−5^; Figure S3). Significant associations were also found for heterozygous (OR = 1.08, 95% CI: 1.01–1.16, P = 0.03) and homozygote (OR = 1.18, 95% CI: 1.07–1.31, P = 0.001). Significant associations were also detected among East Asian populations with per-allele OR of 1.08 (95% CI: 1.03–1.14, P = 0.004), while no significant associations were detect for African and other ethnic populations in all genetic models (Table 3).

**Table 3:** Meta-analysis of the 5p12 rs4415084 polymorphism on breast cancer risk.

Sub-groupanalysis	No. of data sets	No. of cases/controls	T allele				Heterozygous				Homozygous			
			OR (95%CI)	P(Z)	P(Q)^a^	P(Q)^b^	OR (95%CI)	P(Z)	P(Q)^a^	P(Q)^b^	OR (95%CI)	P(Z)	P(Q)^a^	P(Q)^b^
Ethnicity						0.03				0.01				0.15
Caucasian	22	30587/64012	1.10 (1.06–1.14)	<10^−5^	<10^−4^		1.08 (1.01–1.16)	0.03	0.002		1.18 (1.07–1.31)	0.001	<10^−4^	
East Asian	5	6560/6805	1.08 (1.03–1.14)	0.004	0.74		1.04 (0.71–1.50)	0.85	0.004		1.12 (0.96–1.31)	0.14	0.33	
African	3	2790/2901	1.00 (0.87–1.14)	0.96	0.06		0.97 (0.82–1.14)	0.68	0.38		0.99 (0.76–1.30)	0.96	0.11	
Other	2	509/685	1.12 (0.94–1.33)	0.22	0.57		1.18 (0.86–1.63)	0.30	0.33		1.33 (0.88–2.02)	0.18	0.28	

a Cochran’s chi-square Q statistic test used to assess the heterogeneity in subgroups.

b Cochran’s chi-square Q statistic test used to assess the heterogeneity between subgroups.

**Figure 2:**
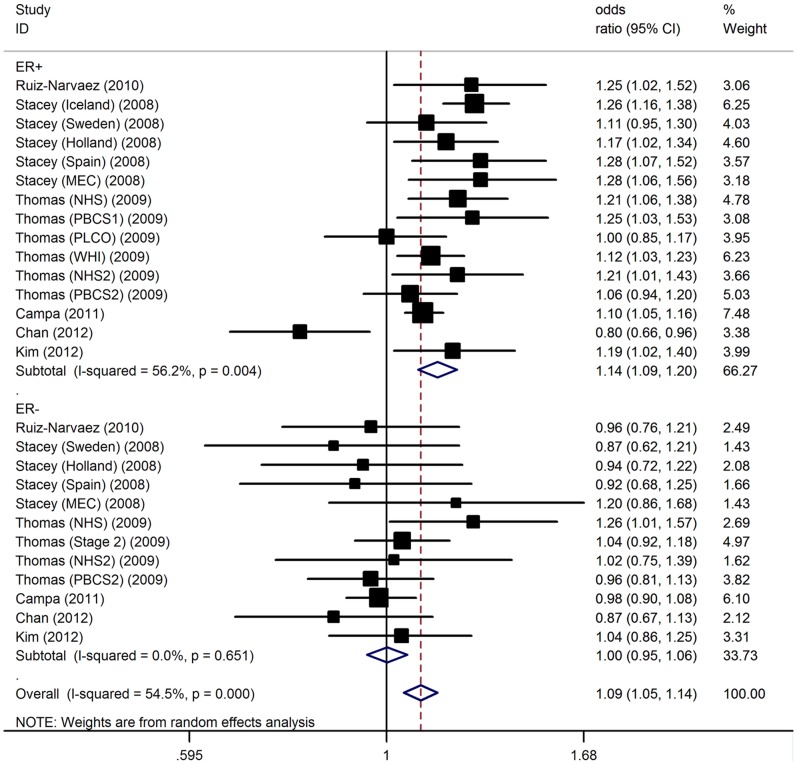
Association between 5p12 rs4415084 and breast cancer risk by ER status.

To investigate whether the polymorphism were associated with particular forms of breast cancer, we analyzed the associations between rs4415084 and breast cancer risk by ER status. The polymorphism was statistically significantly associated with greater risk of ER+ breast cancer [per-allele OR = 1.14, 95% CI: 1.09–1.20; P(Z) <10^−5^; P(Q) = 0.004] than ER- breast cancer [per-allele OR = 1.00, 95% CI: 0.95–1.06; P(Z) = 0.96; P(Q) = 0.65] (Figure 2).

### Association of rs981782 Polymorphism with Breast Cancer

For Caucasian populations, the overall per-allele OR of the T variant for breast cancer was 1.05 (95% CI: 1.02–1.07, P = 0.001; Figure S4), with corresponding results for heterozygous and homozygous of 1.03 (95% CI: 0.98–1.07, P = 0.24) and 1.07 (95% CI: 0.98–1.17, P = 0.12), respectively. However, no significant associations were detected among African in all comparisons (Table 4). No significant between-study heterogeneity was detected for the vast majority of comparisons (Table 4).

**Table 4:** Meta-analysis of the 5p12 rs981782 polymorphism on breast cancer risk.

Sub-groupanalysis	No. of data sets	No. of cases/controls	T allele				Heterozygous				Homozygous			
			OR (95%CI)	P(Z)	P(Q)^a^	P(Q)^b^	OR (95%CI)	P(Z)	P(Q)^a^	P(Q)^b^	OR (95%CI)	P(Z)	P(Q)^a^	P(Q)^b^
Ethnicity						NA				NA				NA
Caucasian	16	43868/77535	1.05 (1.02–1.07)	0.001	0.08		1.03 (0.98–1.07)	0.24	0.43		1.07 (0.98–1.17)	0.12	0.04	
African	1	741/657	1.11 (0.86–1.44)	0.43	NA		0.61 (0.23–1.63)	0.32	NA		0.74 (0.28–1.91)	0.53	NA	

NA: not available.

a Cochran’s chi-square Q statistic test used to assess the heterogeneity in subgroups.

b Cochran’s chi-square Q statistic test used to assess the heterogeneity between subgroups.

### Sensitivity Analyses and Publication Bias

Sensitivity analysis indicated that no single study influenced the pooled OR qualitatively, suggesting that the results of this meta-analysis are stable (data not shown). The shape of the funnel plots was symmetrical for these polymorphisms (Figure S5–S7). The statistical results still did not show publication bias in these studies for rs10941679 (Begg’s test, P = 0.74; Egger’s test, P = 0.53), rs4415084 (Begg’s test, P = 0.76; Egger’s test, P = 0.08) and rs981782 (Begg’s test, P = 0.56; Egger’s test, P = 0.25).

## Discussion

The association between polymorphisms of 5p12 and breast cancer risk had been originally reported by Easton et al [9]. However, the accurate relationship of 5p12 common polymorphisms with breast cancer risk for different ethnic populations was still unknown due to the inconsistent findings [21], [23], [27]–[30], [35]–[37]. This is the first comprehensive meta-analysis, which comprise a total of 131,983 BC cases and 200,314 controls from 24 case–control studies, examining the association of three commonly studied polymorphisms of 5p12 (rs10941679, rs4415084, and rs981782) with breast cancer risk. Our results demonstrated that rs10941679-G allele, rs4415084-T allele and rs981782-T allele is a risk factor for developing breast cancer. Replication of initial genome-wide association findings is considered a gold standard for reporting genotype–phenotype associations. The results from the Michailidou et al. [42] and Zheng et al. [43] study of rs10941679 in 5p12 with breast cancer were in line with that of the present meta-analysis.

Genetic heterogeneity is inevitable in disease identification strategy [44]. We identified ethnicity as a potential source of between-study heterogeneity by subgroup analysis and meta-regression. In the stratified analysis by ethnicity, significant associations were found in Caucasians and East Asians for 5p12-rs10941679 and 5p12-rs4415084 polymorphisms. However, no associations were found in African and other ethnic populations. Similar results were also observed for 5p12-rs981782. There are several possible reasons for such differences. Firstly, the frequencies of the risk-association alleles in these polymorphisms vary between different races. For example, the G allele distribution of the rs10941679 varies between Caucasians, East Asians, and African populations, with a prevalence of 25%, 51%, and 19%, respectively [15], [16], [32], [33]. rs10941679 and rs4415084 are in a region of high LD as reported by Stacey et al [15]. As for rs4415084, the distribution of the risk T allele varies extensively between different races, with a prevalence of more than 40% among Caucasians and East Asians and ∼25% among Africans and other population. Thus, failing to identify any significant association in Africans and other populations could be due to substantially lower statistical power caused by the relatively lower prevalence of T allele. Therefore, additional studies are warranted to further validate ethnic difference in the effect of these polymorphisms on breast cancer risk. Secondly, study design or small sample size or some environmental factors may affect the results. Most of these studies did not consider most of the important environmental factors. It is possible that variation at this locus has modest effects on breast cancer, but environmental factors may predominate in the progress of breast cancer, and mask the effects of this variation. Specific environmental factors like lifestyle and hormone use that have been already well studied in recent decades [3]. In addition, different populations usually have different linkage disequilibrium patterns. A polymorphism may be in close linkage with another nearby causal variant in one ethnic population but not in another. These polymorphisms may be in close linkage with different nearby causal variants in different populations.

Because ER status is one of the major markers of breast cancer subtypes, we further performed analyses to test for differences in the associations of these polymorphisms with breast cancer risk with respect to different ER status. The findings that 5p12-rs10941679 and 5p12-rs4415084 largely affect ER-positive disease are confirmed by our meta-analysis of all available data. The pooled OR estimate is consistent with little or no effect of the two SNPs on ER-negative disease. These findings provide further support for the notion that ER-negative and ER-positive tumors result from different etiologic pathways, rather than different stages of tumor evolution within a common carcinogenic pathway [45]. The magnitude of the observed differences is small, and by themselves these findings are unlikely to have any immediate clinical implications. However, the observed differences provide clues to the biological mechanisms that underpin tumor heterogeneity, which may ultimately lead to improved treatment and prevention.

Large sample and unbiased epidemiological studies of predisposition genes polymorphisms could provide insight into the in vivo relationship between candidate genes and complex diseases. Nevertheless, small sample sized association studies lack statistical power and have resulted in apparently contradicting results. The interpretation of these studies has been further complicated by the use of different ethnic populations and phenotypic heterogeneity. An alternative therefore is to pool data from a range of studies via meta-analysis, thus enhancing the statistical power of the analysis for the estimation of genetic effects. The familial excess in risk not accounted for by *BRCA1* or *BRCA2* is plausibly explained by a polygenic model in which a large number of “low-penetrance” variants act in combination to cause wide variation in risk in the population [46]. Recently, several “low-penetrance” variants have been identified through meta-analysis, such as *FGFR2* [47], *XRCC1* [48], and *SLC4A7* [49] . These studies greatly contribute to the understanding of the genetic as well as the pathological basis of the development of breast cancers.

Several potential limitations of the present meta-analysis should be taken into consideration. Firstly, the subgroup meta-analyses dealing with interactions between the 5p12 polymorphisms and Africans or other ethnic populations are based on the small number of studies where such information is available. As studies among the African or other ethnic populations are currently limited, further studies including a wider spectrum of subjects should be carried to investigate the role of these variants in different populations. Secondly, our results were based on unadjusted estimates, while a more precise analysis should be conducted if all individual raw data were available, which would allow for the adjustment by other co-variants including age, cigarette consumption, alcohol drinking, menopausal status, and other lifestyle. Thirdly, the single locus–based nature of meta-analysis precluded the possibility of gene-gene and gene-environment interactions, as well as haplotype-based effects, suggesting that additional studies assessing these aspects are necessary.

In summary, findings from this meta-analysis indicate that 5p12 rs10941679, rs4415084, and rs981782 polymorphism is significantly associated with an increased risk of breast cancer, particularly in Caucasian and East Asian populations. More work is needed to further investigate the association of these polymorphisms across different histological types or tumor staging of breast cancer. Besides, future studies are recommended to identify the possible gene-gene and gene-environmental interactions in this association.

## Supporting Information

Figure S1 Study selection process.
(TIF)

Figure S2 Forest plot from the meta-analysis of breast cancer risk and 5p12 rs10941679 polymorphism.
(TIF)

Figure S3 Forest plot from the meta-analysis of breast cancer risk and 5p12 rs4415084 polymorphism.
(TIF)

Figure S4 Forest plot from the meta-analysis of breast cancer risk and 5p12 rs981782 polymorphism.
(TIF)

Figure S5 Begg’s funnel plot for publication bias in studies on 5p12-rs10941679 polymorphism and breast cancer.
(TIF)

Figure S6 Begg’s funnel plot for publication bias in studies on 5p12-rs4415084 polymorphism and breast cancer.
(TIF)

Figure S7 Begg’s funnel plot for publication bias in studies on 5p12-rs981782 polymorphism and breast cancer.
(TIF)

Checklist S1 (DOC)
