# Supplementary figures and images for: Quantitative Assessment of Common Genetic Variants on Chromosome 5p12 and Hormone Receptor Status with Breast Cancer Risk
Source: PLoS One. 2013 Aug 19;8(8):e72154. doi: 10.1371/journal.pone.0072154 (PMC3747047; doi:10.1371/journal.pone.0072154)

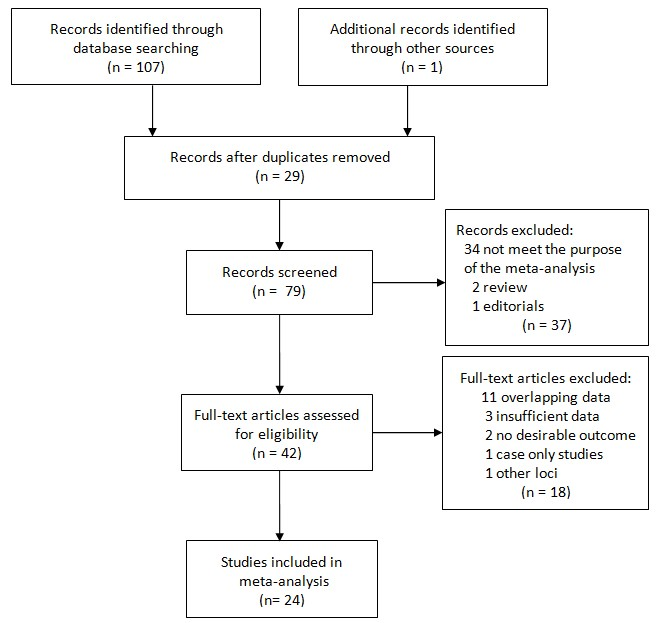

Supplement: Figure S1 — Study selection process. (TIF) [file pone.0072154.s001.tif]

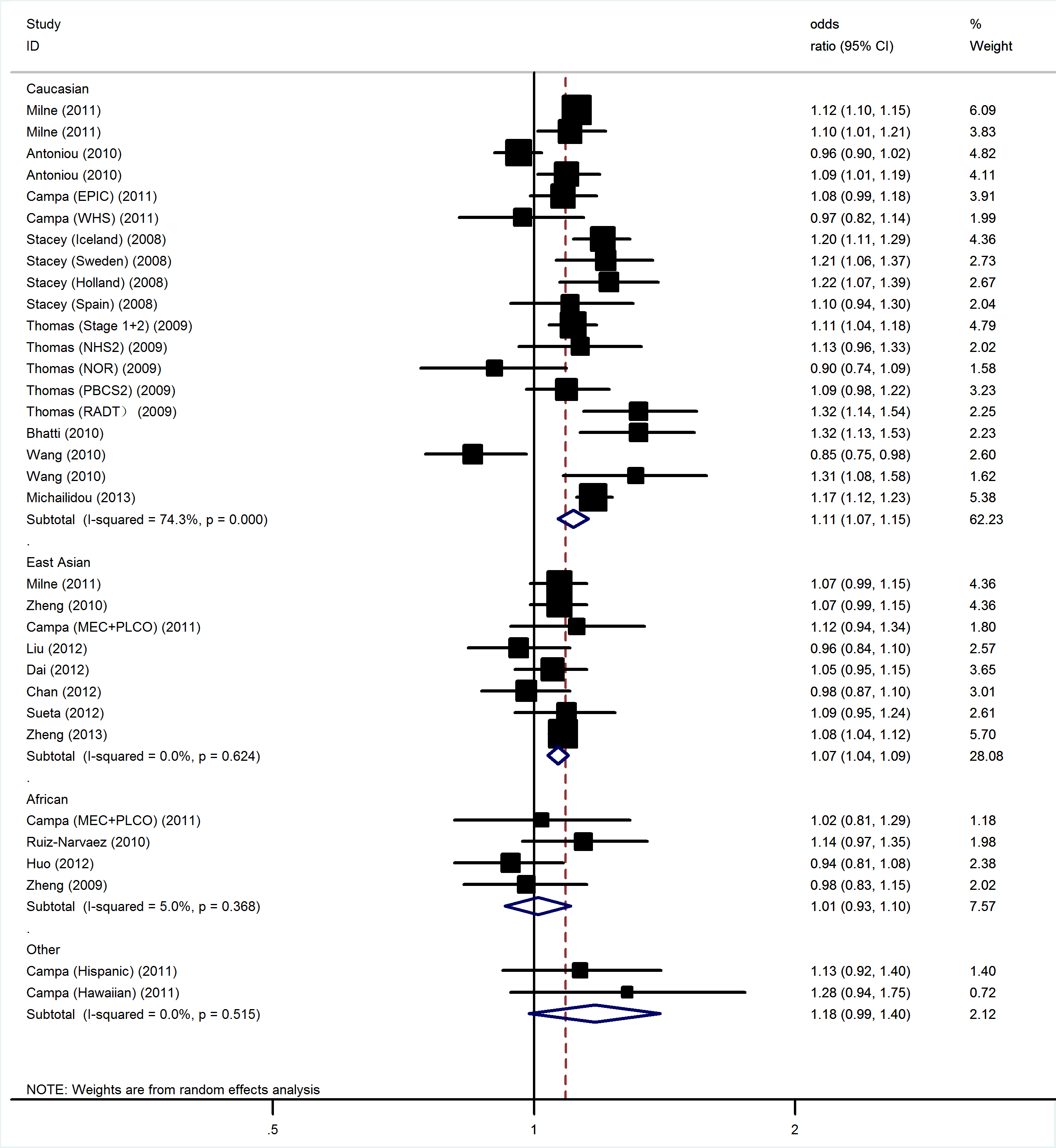

Supplement: Figure S2 — Forest plot from the meta-analysis of breast cancer risk and 5p12 rs10941679 polymorphism. (TIF) [file pone.0072154.s002.tif]

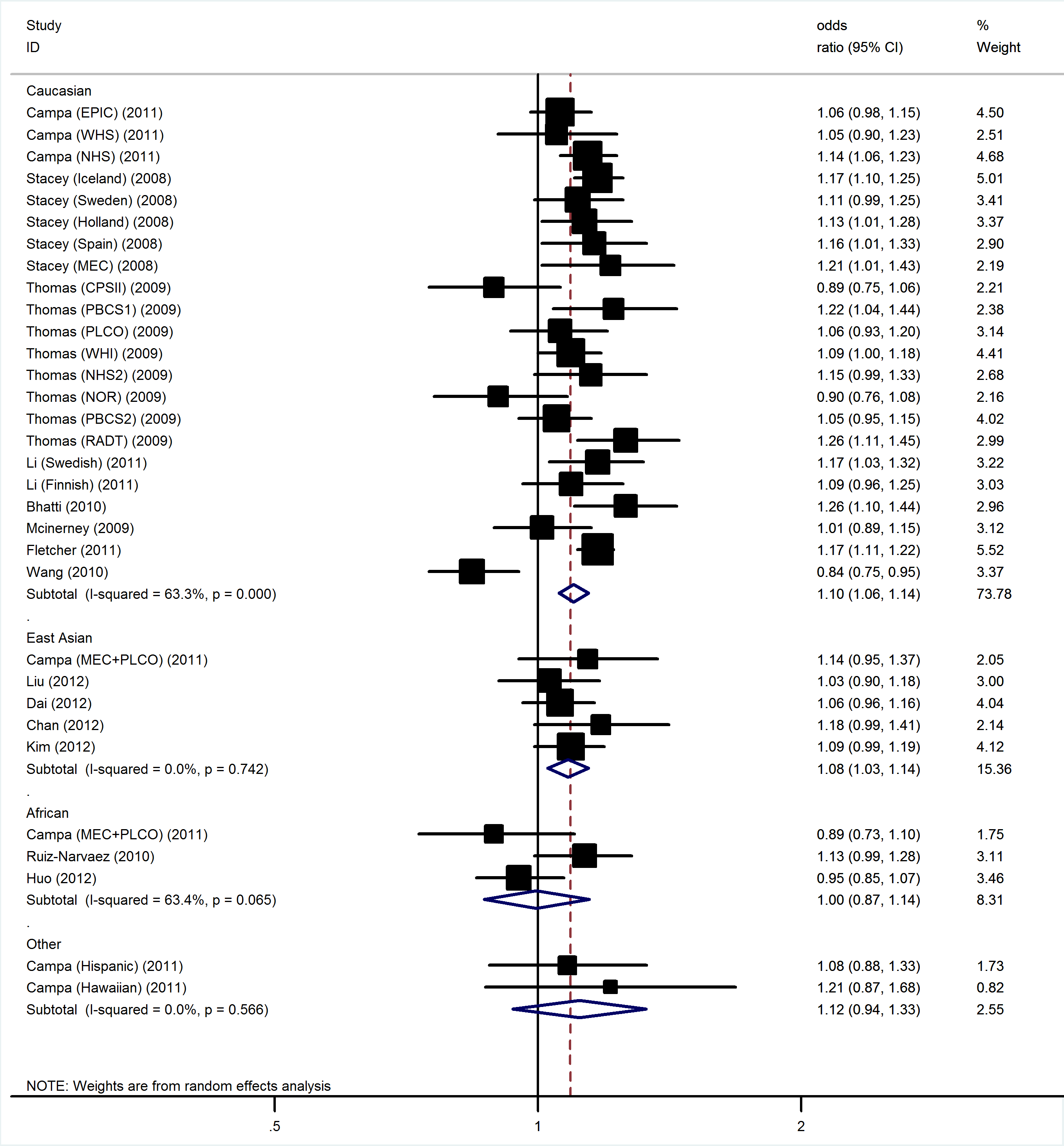

Supplement: Figure S3 — Forest plot from the meta-analysis of breast cancer risk and 5p12 rs4415084 polymorphism. (TIF) [file pone.0072154.s003.tif]

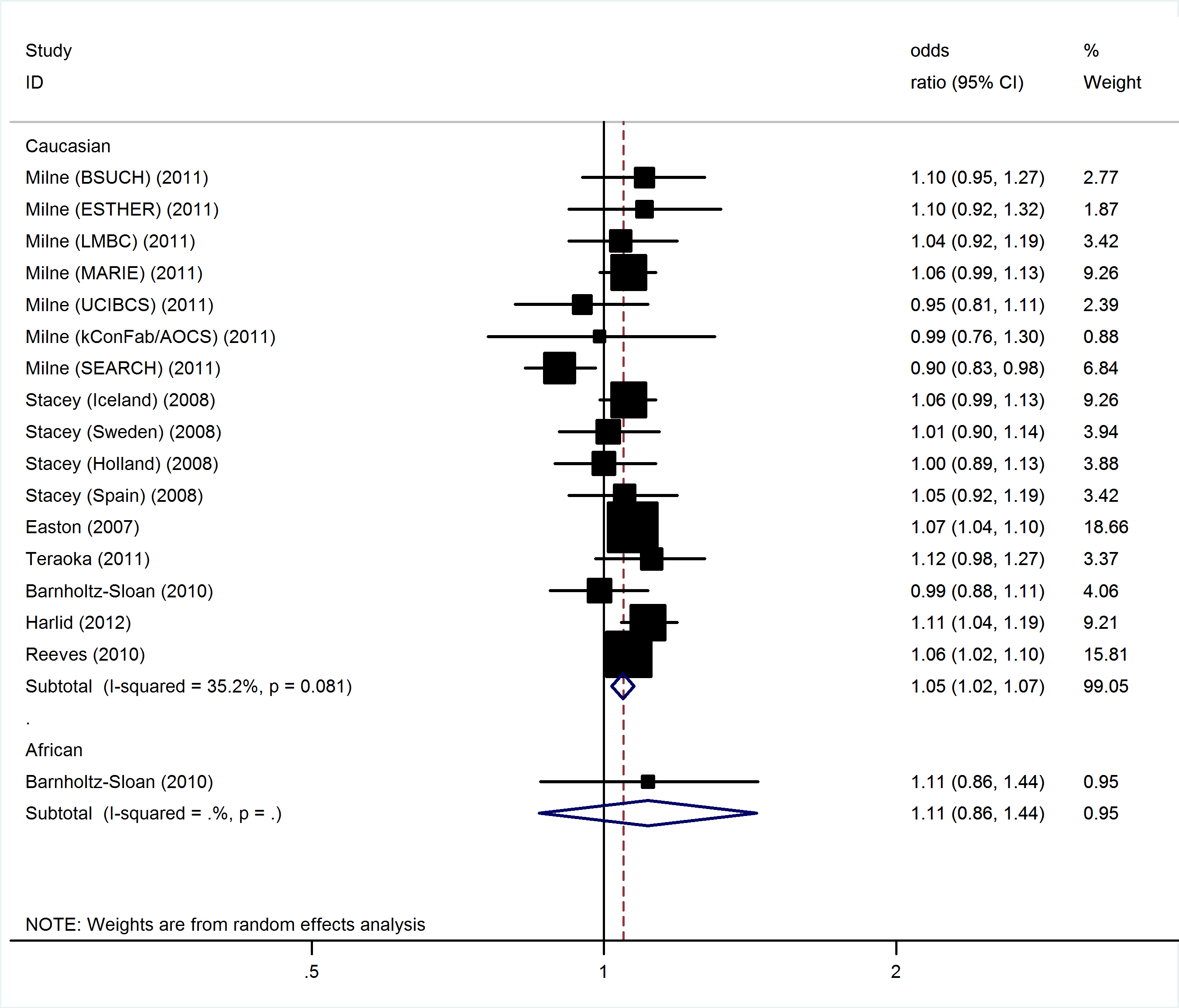

Supplement: Figure S4 — Forest plot from the meta-analysis of breast cancer risk and 5p12 rs981782 polymorphism. (TIF) [file pone.0072154.s004.tif]

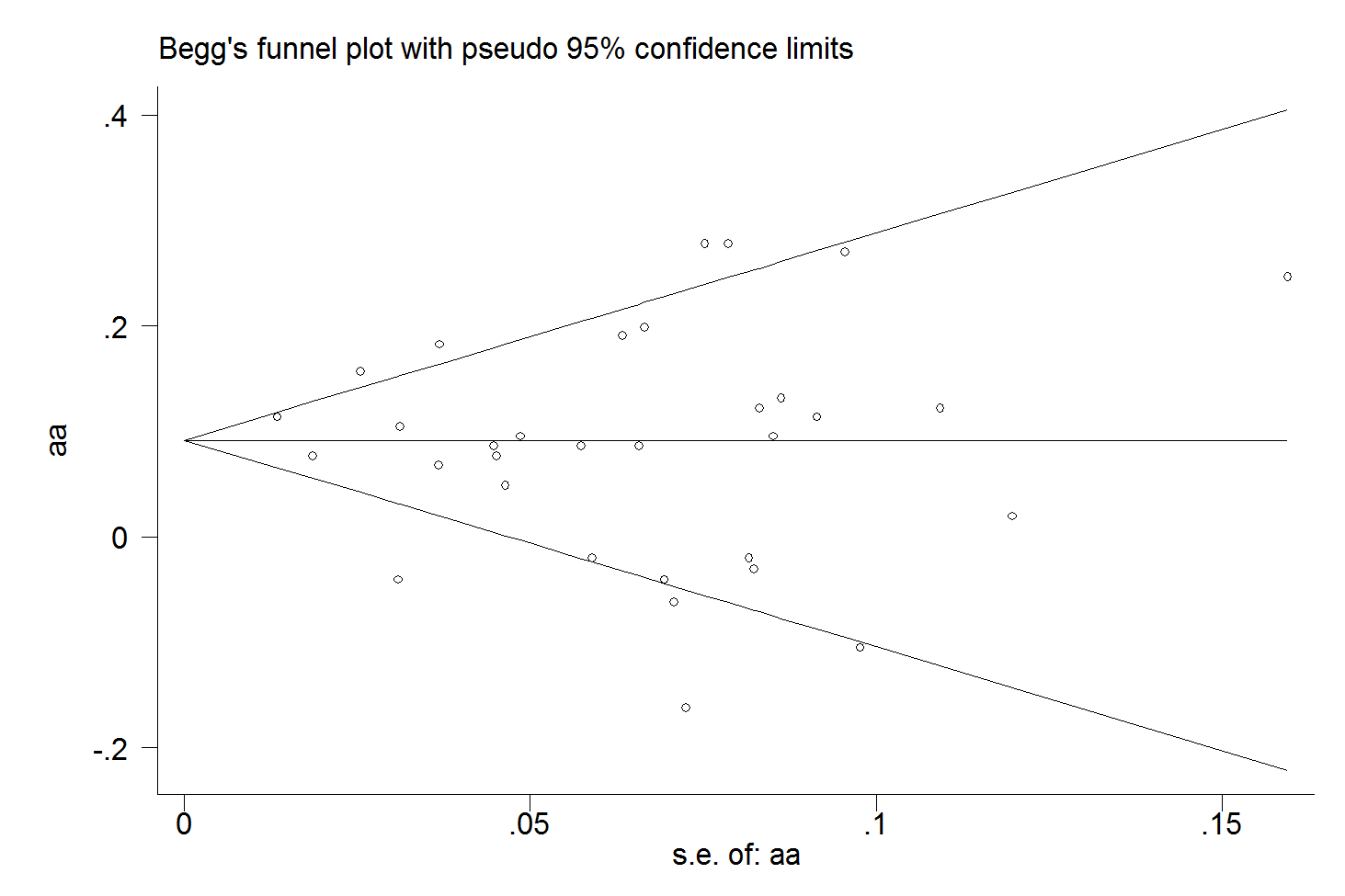

Supplement: Figure S5 — Begg’s funnel plot for publication bias in studies on 5p12-rs10941679 polymorphism and breast cancer. (TIF) [file pone.0072154.s005.tif]

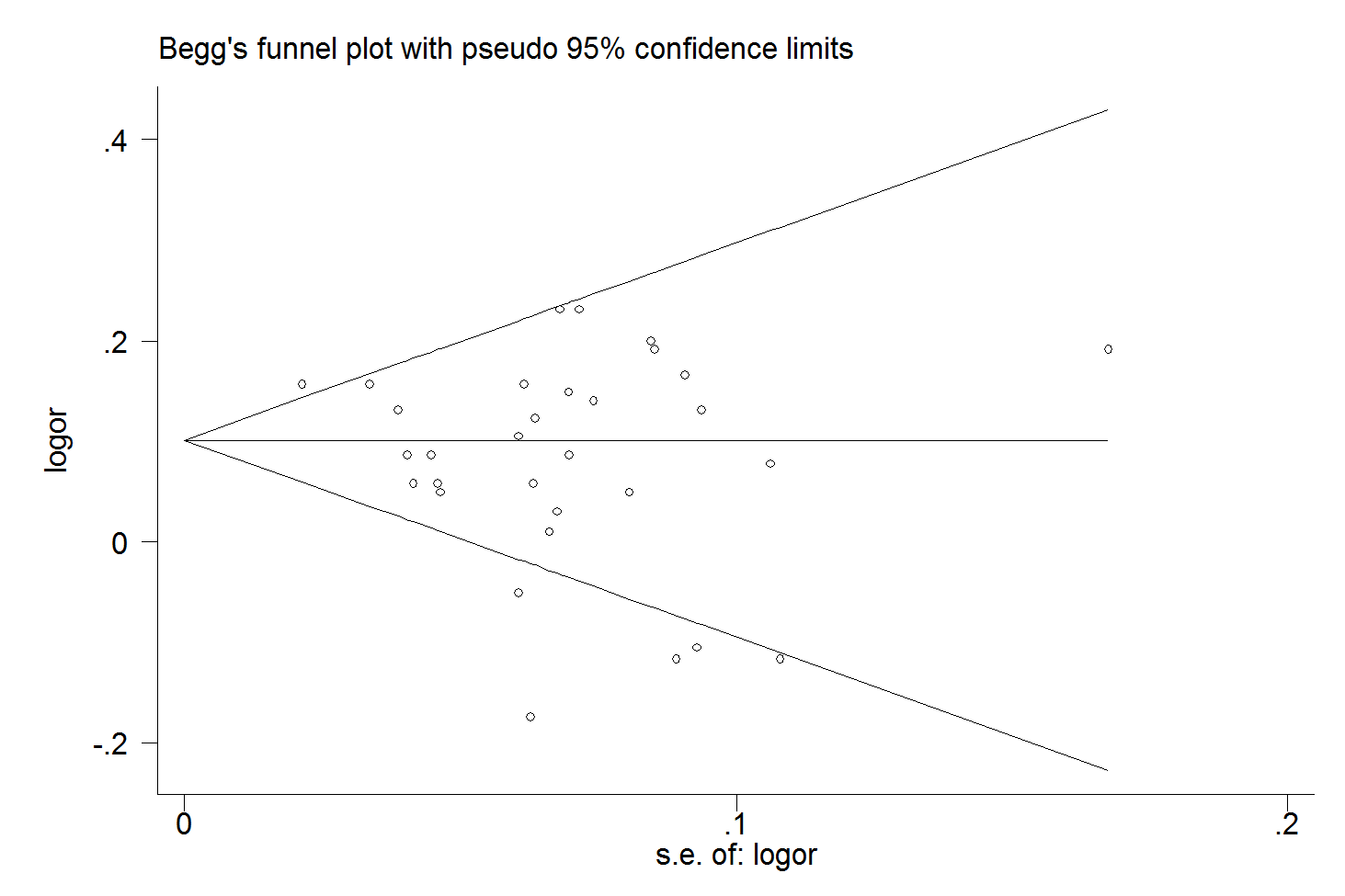

Supplement: Figure S6 — Begg’s funnel plot for publication bias in studies on 5p12-rs4415084 polymorphism and breast cancer. (TIF) [file pone.0072154.s006.tif]

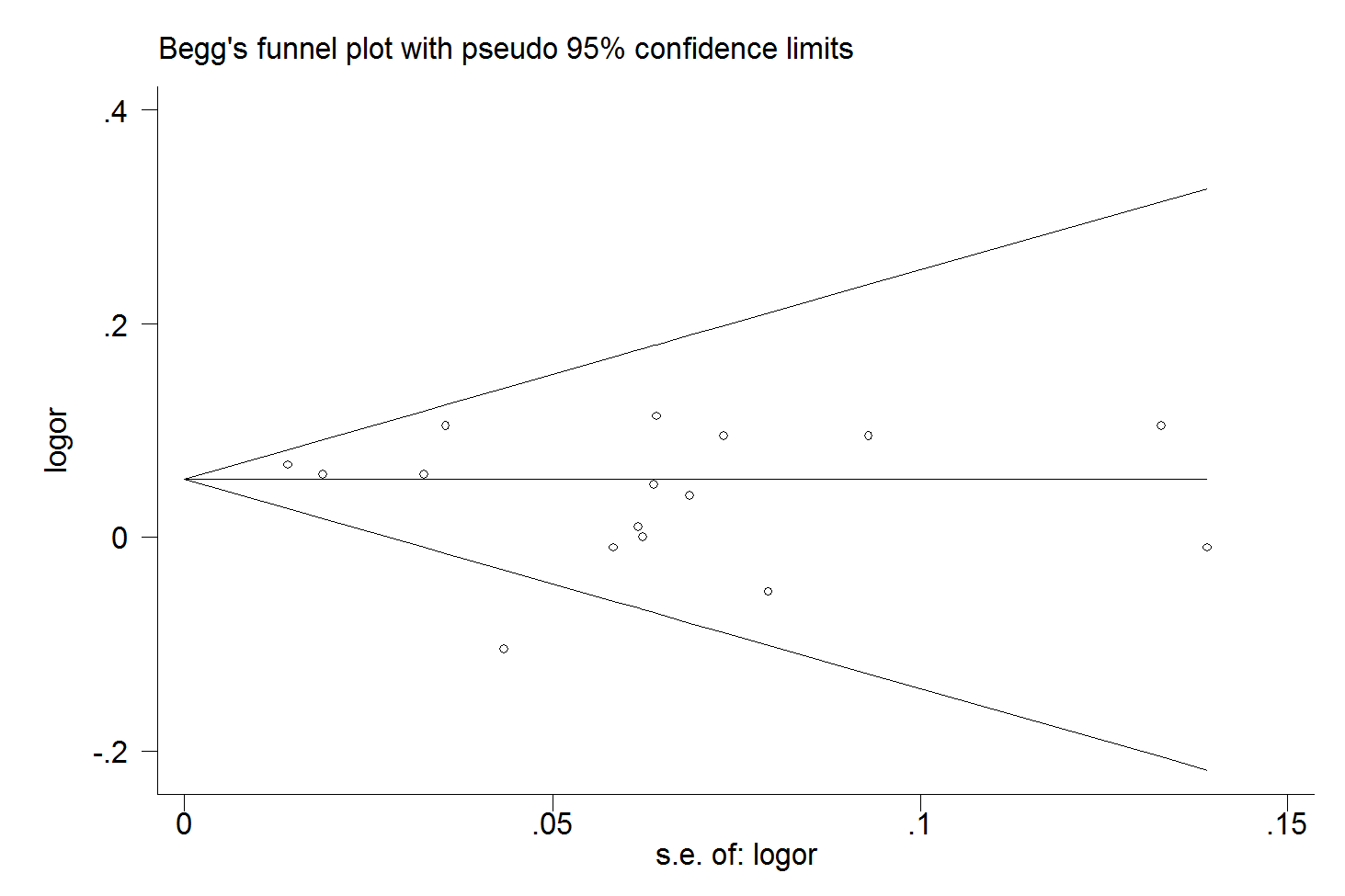

Supplement: Figure S7 — Begg’s funnel plot for publication bias in studies on 5p12-rs981782 polymorphism and breast cancer. (TIF) [file pone.0072154.s007.tif]
